# Evaluation of Plasma Amino Acid Levels in Preterm Infants and Their Potential Correlation with Retinopathy of Prematurity

**DOI:** 10.1155/2020/8026547

**Published:** 2020-11-10

**Authors:** Yasin Ozcan, Gumus Huseyin, Kenan Sonmez

**Affiliations:** ^1^Yeditepe University, Faculty of Medicine, Ophthalmology Department, Istanbul, Turkey; ^2^Harran University, Medical School Hospital, Neonatology Department, Sanliurfa, Turkey; ^3^University of Health Sciences, Ulucanlar Eye Education and Research Hospital, Ophthalmology Department, Ankara, Turkey

## Abstract

**Aim:**

The aim of this study is to ascertain whether the level of circulating amino acids (aa) is associated with retinopathy of prematurity (ROP).

**Methods:**

This is a randomized controlled study of 55 infants born at gestational age (GA) ≤32 weeks or birth weight (BW) ≤1500 grams. Serum samples were obtained from two groups: Group A comprised of 26 preterm infants with ROP and Group B comprised of 29 preterm infants without ROP. Plasma aa levels were analyzed using liquid chromatography with tandem mass spectrometry (LC-MS/MS). Correlation test and multivariate regression analysis were used to evaluate the relationship between plasma aa levels and variables.

**Results:**

The mean serum arginine and glutamine levels were significantly higher, but the mean lysine and aspartic acid levels were significantly lower in Group A, compared to Group B (*p* = 0.04, *p* = 0.002, *p* = 0.029, and *p* = 0.002, respectively). In multivariate analysis, the mean arginine and lysine levels were significantly associated with the stage of the disease (*p* = 0.03 and *p* = 0.01, respectively). No significant differences were determined between the groups in terms of alanine, asparagine, valine, leucine, phenylalanine, tyrosine, serine, proline, citrulline, cysteine, ornithine, tryptophan, methionine, threonine, taurine, and isoleucine amino acids (*p* > 0.05, respectively).

**Conclusions:**

These results indicate a significant association between high arginine and glutamine, with low lysine and aspartic acid serum concentrations with ROP. Due to higher serum concentrations in ROP, extra arginine-glutamine supplementation in hyperoxic conditions may be unfavorable through pathways involving reactive oxygen, particularly in patients with ROP.

## 1. Introduction

Retinopathy of prematurity (ROP) is a preventable, sight-threatening condition associated with abnormal retinal vascular development that occurs only in premature infants [1]. Risk factors for the development of ROP can be summarized under two main headings as prenatal factors, such as low gestational age at birth and low birth weight, and postnatal factors such as exposure to higher oxygen levels and loss of fetomaternal interaction. These factors lead to an increase in metabolic demands and a reduction in the production of insulin-like growth factor-I (IGF-I), which is a key factor for body growth and weight gain in postnatal period [2]. Poor general growth and low serum IGF-1 concentrations are associated with neonatal morbities such as ROP, intraventricular hemorrhage (IVH) and necrotizing enterocolitis (NEC) [3]. The synthesis of IGF-I is regulated by the availability of amino acids (AA) and depends on overall energy intake; therefore, circulating AA concentrations in preterm babies might be linked with ROP by downregulation of IGF-1 synthesis. However, the exact role of serum amino acids on the underlying mechanism of ROP remains unknown [3].

Early interruption of fetal-maternal interaction may lead to a deficiency of nutrients that are essential to maintain the normal vascularization process. In a mice model, it was shown that Vitamins C, E, and omega-3 fatty acid supplementation improved retinal vascularization in phase 1 oxygen-induced retinopathy model, and the administration of dipeptide arginyl-glutamine in phase 2 diminished retinal vasoproliferation by reducing vascular endothelial growth factor (VEGF) expression, thereby suggesting that AA deprivation might be considered to contribute to oxygen-induced retinopathy [4–6]. Exogenous factors including oxidative stress, inflammation, and nutritional capacity have been found to be linked to severe ROP through different mechanisms, based on animal model studies [7].

Early aggresive parenteral nutrition with AAs and lipids is associated with higher levels of IGF-1 and IGFBP-3 which might decrease the risk of retinopathy of prematurity [8]. Stress, commonly seen in premature infants in neonatal intensive care units, induces amino acid deprivation, particularly arginine and glutamine deficiency. Arginine regulates nitric oxide pathways that may produce nicotinamid adenine dinucleotide phosphate (NADPH) oxidase-mediated reactive oxygen species such as peroxynitrit. It may cause endothelial vascular damage, which contributes to the progression of phase 1 and phase 2 ROP in experimental models [9, 10].

The purpose of this study was to ascertain whether abnormal serum AA concentrations may accompany with the presence of ROP and sought to find the association between serum AA levels and pathogenesis of premature retinopathy, which is strongly associated with high oxygen exposure.

## 2. Subjects and Methods

### 2.1. Study Design

This case-control randomized study was conducted from March 2018 to December 2018 in a tertiary referral center for retinopathy of prematurity (ROP) and a university hospital. Informed consent was obtained from the parents of all participants prior to examination and any measurements were taken for each infant before enrollment. The study was approved by the Institutional Review Board/Ethics Committee of Harran University with the registration number on a public trials registry, 4059997–050.04.04, in Turkey and was carried out in accordance with the Declaration of Helsinki.

### 2.2. Patient Selection

Infants with birth weight (BW) ≤1500 grams or gestational age ≤32 weeks (GA) and prematurity with BW > 1500 grams or GA > 32 weeks with a history of admission to a neonatal intensive care unit (NICU) due to an unstable clinical course were eligible to participate in this study. The admission criteria for NICU consist of prematures having respiratory distress requiring ventilatory support, seizures, congenital heart disease, or cardiac arrhythmias requiring cardiac services, with severe hypoxic–ischemic injury, birth weight between 1000 grams and 2000 grams, gestational age between 32 and 36 weeks, prematurity and/or birth weight of less than 1500 g, and severe intrauterine growth restriction [11].

Due to the fact that Bas et al. [12] reported that 4.1% of prematures with >35 weeks GA had developed any stage of ROP in NICU, particularly in our country, the cut-off value was determined as ≥36 weeks GA in terms of inclusion criteria for the study.

A total of 55 premature infants were included and assigned into 2 groups based on the presence of ROP. Twenty-six premature infants with ROP were defined as Group A and 29 prematures without ROP were defined as Group B. Prematures, requiring consecutive screening examinations for ROP arranged by international guideline [13], who received same nutrition protocol, and regularly continued to their follow-up examination, having a stable clinical condition which allows us to perform an eye examination, were included in the study. The exclusion criteria were having a history of previous eye surgery, metabolic disease, failure of any organ, lack of cooperation by the family, discontinuation of the follow-up period, patients in the NICU who received a different nutrition protocol of partial enteral and/or parenteral nutrition treatment to maintain safe plasma levels of amino acids and supply the required daily calories, and participants with any history of previous admission to NICUs for receiving oxygen supplementation therapy along with parenteral/enteral nutrition or requiring any other medical intervention in order to stabilize the clinical status.

### 2.3. Nutrition Protocol

All infants received the same nutrition protocol, which followed international principles [14, 15]. The nutrition protocol used in the study has not been designed specifically for the study. It has been in use in NICU for many years. In the first week of life, premature infants were fed with total parenteral nutrition (TPN) 2 g/kg/per day, corresponding to 60–70 kcal/day. In the second week of life, the amount of TPN was increased to 3 g/kg/per day. In the third week of life, feeding with breast milk and the same formula was added to TPN, corresponding to 100–120 kcal daily. In the fourth week, the infants were fed with breast milk only and the same formula.

### 2.4. Ophthalmological Examination

Ophthalmological examinations were performed following dilatation of the pupils (with a 0.5% tropicamide and 1.0% phenylephrine solution). Instruments were used in sterile package forms, including an eyelid speculum and scleral depressor. A +28 diopter lens and indirect ophthalmoscope were used for visualization of the retina. A nurse was available to swaddle the infant and stabilize the head as well as to intervene if the infant experiences apnea or bradycardia during the examination [16].

The international guidelines were followed in the screening examination for ROP. The initial examination was performed at 4 to 6 weeks' postnatal age or 31 weeks' postmenstrual age. After each examination, the follow-up interval is determined based on the presence or absence of ROP and the disease features. When any stage of ROP was detected, examinations were repeated at weekly or biweekly intervals depending on the retinal findings until reaching a fully vascularized peripheral retina or complete resolution of ROP was obtained [13].

An ophthalmologist experienced in the screening and treatment of ROP performed all the examinations (YO). The retinal examination findings were classified according to the current International Classification of ROP. The classification defines the disease by location or the retinal zone of involvement, severity, stage or extent of the disease in clock hours, and whether or not plus disease is present. Plus disease refers to increased arteriolar tortuosity and venous engorgement of the retinal vasculature, indicating the severity of ROP [16].

### 2.5. Blood Sampling and Laboratory Analysis

The levels of 19 amino acids were analyzed in blood samples derived from ROP patients and premature infants without ROP. Blood samples were collected after the initial eye examination and before feeding by the pediatricians in the outpatient clinic. The quantitation of amino acids from plasma was achieved using liquid chromatography with tandem mass spectrometry (LC-MS/MS) as described previously [17]. For the analysis of serum amino acids, 2 mL venous blood samples were taken in heparinized tubes and centrifuged. After 50 *μ*l of centrifuged plasma was taken up in the tube (Eppendorf AG, Hamburg, Germany), 50 *μ*l amino acid ISTD (internal standard) was added and vortexed for 5 seconds. In the final step, 700 *μ*l of R-1 (Reagent 1) was added and vortexed again for 5 seconds and then centrifuged at 10000 rpm for 10 minutes. After taking the supernatant into the vial tube, the triple quadripole SHIMADZU LC-MS/MS 8045 was included in the assay. Standard concentrations were in the range of 31–92 *μ*mol/L for arginine, 14–114 *μ*mol/L for lysine, and 0–25 *μ*mol/L for aspartate. The internal standard contained alanine, arginine, asparagine, valine, leucine, isoleucine, lysine, phenylalanine, glutamine, glutamate, tyrosine, serine, proline, citrulline, cysteine, ornithine, tryptophan, methionine, and threonine amino acids.

Only one blood sample was taken for identifying amino acid concentrations for each patient after the first ROP examination. Following eye examination, the files of all participants with and without ROP were checked in terms of having received the same nutrition protocol in order to decide whether the patient is suitable for blood sampling.

### 2.6. Statistical Analysis

All statistical analyses of the study were performed using SPSS for Windows (SPSS Inc., Chicago, IL, USA) software. The Kolmogorov–Smirnov test was used to assess the conformity of data to normal distribution. All data obtained from the two groups were expressed as mean ± standard deviation (SD). The Student's *t-*test and Mann–Whitney-*U* tests were used to compare birth weight, gestational age, and the levels of amino acids between the two groups. Correlations between variables were determined using Pearson's and Spearman's tests. When a significance result was obtained, multivariate regression analysis was used to evaluate the associations between the aa levels and GA and BW for each group. In addition, the relationship between the significant variables and the stage of disease was investigated through this test. A value of *p* < 0.05 was considered statistically significant.

## 3. Results

The 55 premature infants were separated into Group A (26 with ROP) and Group B (29 without ROP). The mean gestational age (GA) was 28.5 ± 2.7 weeks and the mean birth weight (BW) was 1325.2 ± 367 grams in Group A. The mean GA was 31.52 ± 2.6 weeks and the mean BW was 1351.3 ± 308 grams in Group B. The mean GA was significantly lower in Group A and no significant difference was found between the groups in terms of BW (*p* < 0.05, *p* = 0.2, respectively).

The mean body weight measured at the time of blood sampling, the mean days in NICU, the mean follow-up period, and the associated *p* values are summarized in Table 1.

The mean arginine and glutamine levels were significantly higher, but the mean lysine and aspartic acid levels were significantly lower in Group A, compared to Group B (*p* = 0.04, *p* = 0.002, *p* = 0.029, and *p* = 0.002, respectively). Comparisons of the mean values and statistical analyses of the serum amino acid samples of the groups are analyzed in Table 2.

In Group A, 13 patients (50%) had stage 1 and 12 patients had stage 2 (46%) of ROP without any sign of plus.

**Figure 1:**
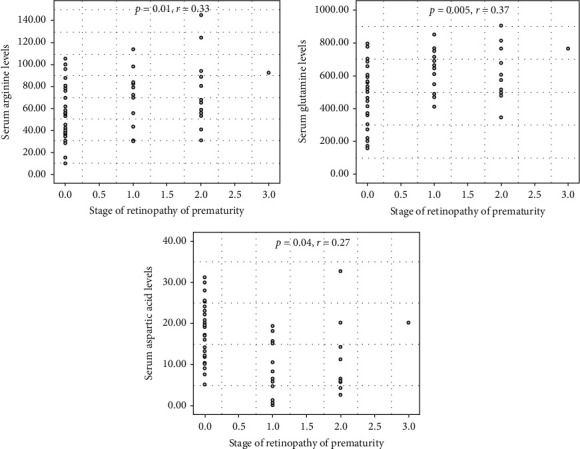
The scatter plots indicate a significant correlation between the stage of ROP and serum arginine, glutamine, and aspartic acid levels.

In group A, there was a significant positive correlation between the stage of ROP and the mean arginine and glutamine levels, although a significant negative correlation was found between the stage of ROP and the mean aspartic acid level (*p* = 0.01, *r* = 0.33; *p* = 0.005, *r* = 0.37, and *p* = 0.04*r* = −0.27, respectively) (Figure 1).

In multiple regression analysis, the mean arginine, lysine, aspartic acid, and glutamine levels were not associated with GA and BW in Group A (for GA; *p* = 0.79, *p* = 0.20, *p* = 0.36, and *p* = 0.05; for BW; *p* = 0.41, *p* = 0.12, *p* = 0.43, and *p* = 0.29, respectively).

## 4. Discussion

The present study showed that the mean plasma arginine and glutamine levels were significantly higher in premature infants with ROP, compared to prematures without ROP. In addition, the correlation between serum arginine and glutamine levels and the stage of disease was found to be statistically significant. Consistent with the findings of the present study, Posod et al. [18] reported that significantly higher plasma concentrations of arginine, glutamine, citrulline, and tryptophan, indicating a dualistic cardiovascular risk profile, were observed in preterm infants. However, in contrast to these findings, Neu et al. [19] previously demonstrated that the administration of dipeptide arginine and glutamine (Arg-Gln) dramatically inhibited retinal neovascularization by decreasing vascular endothelial growth factor (VEGF) mRNA levels in retinal tissue in an oxygen-induced retinopathy (OIR) model. Similarly, Shaw et al. [20] showed a significant reduction in pre-retinal neovascularization and vaso-obliteration in pups that received dipeptide Arg-Gln supplementation in an OIR model through the restoration of retinal docosahexaenoic acid (DHA) and neuroprotectin D1 levels.

Arginine plays a vital role in fetal and neonatal growth and development, serving as a physiological precursor for the synthesis of crucial molecules, including nitric oxide (NOX), creatine, urea, glutamate, and polyamines [9]. Glutamine is an essential amino acid for the metabolic function of cells. Glutamine is used for the synthesis of nicotinamide adenine dinucleotide phosphate (NADPH) and antioxidants, and many pathways involved in the maintenance of cellular integrity and function [21].

NOX is the endothelium-derived relaxing factor, a neurotransmitter, and a signal transduction molecule, which is activated by nitric oxide synthase (NOS)-derived nitric oxide (NO), and has vasoprotective functions that are compromised in the vasodegenerative phase of ROP [10, 22]. Endothelial nitric oxide synthase (e-NOS) can lead to relaxation in the endothelial cells of blood vessels, but at high oxygen concentrations, NOX can transform into nitro-oxidative forms such as peroxynitrite, which result in microvascular degeneration in phase 1 oxygen-induced retinopathy (OIR) models [10].

Nicotinamid adenine dinucleotide phosphate (NADPH) oxidase-generated reactive oxygen species can also cause endothelial cell injury and avascular retina in phase 1 OIR through the activation of isoforms NOX1 or NOX2, or can increase vasoproliferation in phase 2 OIR through the activation of the isoforms, NOX1, NOX2, and NOX4 [9, 23]. Thus, we hypothesized that extra administration of arginine and glutamine may not be beneficial in patients with ROP, as it might increase retinal vasoproliferation and the degenerative process through NOX and reactive oxygen metabolites, particularly at higher O_2_ levels. Todoroki et al. [24] demonstrated that the increased arginine levels had the cytotoxic effect of NB9 human neuroblastoma cells (NB9) by decreasing nitric oxide synthase (nNOS) levels causing the production of reactive oxygen species such as NADPH. However, the favorable effect of arg-gln administration on diminishing retinal neovascularization in experimental OIR models has been shown in different studies [18, 19].

In the current study, the mean plasma lysine (Lys) and aspartate (Asp) amino acid levels were significantly lower in premature infants with ROP. In patients with ROP, there was a significant positive correlation between the stage of ROP and the mean arginine and glutamine levels, whereas a significant negative correlation was found between the stage of ROP and the mean aspartic acid level. In multiple regression analysis, the mean arginine, glutamine, lysine, and aspartic acid levels were not significantly associated with GA and BW in patients with ROP. Lys is an essential, while Asp is a nonessential amino acid. Lys is a common limiting amino acid in human and animal diets and plays an important role in cell proliferation and metabolism. All preterm infants face a period of compromised enteral intake in their first weeks of life due to their immature gastrointestinal tract. They need to receive a combined parenteral and/or enteral nutrients supply to provide a higher energy intake [25, 26]. The intake of essential amino acids required to maintain nitrogen equilibrium in children and infants has been defined as the amount necessary to obtain growth and nitrogen balance [27]. Although amino acid deprivation has been associated with stress occurring in the NICU, the main reason for amino acid deficiencies and the effect on metabolic pathways in premature infants is still unclear. Some studies related to premature infants have reported that plasma amino acid concentrations in premature infants were either lower or higher than the minimum reference values based on the study design. In addition, they indicated that plasma amino acid concentrations did not show a significant difference in preterm infants fed with different human milk diets [17, 28]. The role of Lys and Asp amino acid levels on the mechanism of the development of ROP is unknown.

In this study, the mean body weight at 31 weeks of life, at the time of blood sampling, was not found to be significantly different between the groups. In patients with ROP, 50% of patients had stage 1, 46% of patients had stage 2 disease without plus. All eyes with stage-1 and stage-2 disease regressed spontaneously and completed their peripheral retinal vascularization without requiring any intervention. Only one patient had progressed to stage 3 ROP disease with plus which was treated with diode laser photocoagulation.

The strength of the study was the randomized design distinguishing this from other studies related to the effect of amino acids on the pathogenesis of retinopathy of prematurity. Previous studies of ROP that have investigated amino acids have been experimental, and they have assessed the effect of amino acid administration on ROP following the establishment of a ROP model in animals . The main limitation of this study is that amino acid concentrations were only determined at a single time-point in the neonatal period. Whether alterations would occur with medical interventions or adaptations in perinatal care was not examined. Another limitation of this study was the size of our cohort, which was not very large.

In conclusion, these results indicate an association between the serum levels of high arginine and glutamine, and low lysine and aspartic acid with ROP. The mean serum levels of these amino acids did not have a significant effect on the GA and BW. It is hoped that this study will engender new discussion in literature and encourage further studies to be undertaken to better understand the relationship between the amino acid levels and ROP before considering extra administration of arginine to patients with ROP. Due to higher serum concentrations in ROP, extra arginine-glutamine supplementation in hyperoxic conditions may be unfavorable through pathways involving reactive oxygen, particularly in patients with ROP. Further prospective studies with larger cohorts are needed to clarify whether changes in amino acid dynamics are causally linked to ROP.

## Figures and Tables

**Table 1:** The mean body weights at 31 weeks of life, days in NICU, and follow-up periods and *p* values.

	Group A	Group B	*p* value
Body weight at 31 weeks of life/grams	1669 ± 323	1739 ± 413	*0.16*
Days in NICU/days	21 ± 3	19 ± 5	0.2
Follow-up period/weeks	17 ± 3	11 ± 2	*0.03*

**Table 2:** The analysis of serum amino acid levels and *p* values in both groups.

Amino acids (*μ*mol/L)	Group A (*n* = 26)	Group B (*n* = 29)	Reference values	*p* value
Taurine (Tau)	59.8 ± 23	71 ± 48	>26 – < 130	*0.274*
Tryptophan (Try)	53.2 ± 16	55.4 ± 22	>16 – < 92	*0.673*
Phenylalanine (Phe)	49.5 ± 15	55.5 ± 13	>31 – < 92	*0.133*
Tyrosine (Tyr)	86.7 ± 39	79.5 ± 32	14 – 114	*0.459*
Leucine (Leu)	109.3 ± 46	117.9 ± 40	>43 – < 181	*0.467*
Methionine (Met)	41.6 ± 15	42.3 ± 14	>12 – < 55	*0.864* ^*∗*^
Isoleucine (Ile)	7.5 ± 0.7	8.6 ± 2	28 – 92	*0.065*
Asparagine (Asn)	61.8 ± 35	55.3 ± 23	>20 – < 77	*0.443*
Proline (Pro)	222.4 ± 110	198 ± 98	>104 – < 348	*0.396*
Citrulline (Cit)	30.5 ± 13	25.8 ± 13	>4 – < 50	*0.214*
Cysteine (Cyst)	0.4 ± 0.3	0.4 ± 0.3	>0 – < 1	*0.872* ^*∗*^
Arginine (Arg)	70 ± 30	53.8 ± 25.7	>30 – < 147	*0.041*
Ornithine (Orn)	92.8 ± 37	103.1 ± 44	>19 – < 139	*0.364*
Lysine (Lys)	166.8 ± 67	216 ± 93	>70 – < 258	*0.029*
Valine (Val)	140.1 ± 64	125.7 ± 40	>84 – < 354	*0.333* ^*∗*^
Threonine (Thr)	179.1 ± 73	207.3 ± 89	>40 – < 428	*0.205*
Serine (Ser)	128.4 ± 52	134.5 ± 53	>83 – < 212	*0.674*
Alanine (Ala)	269.9 ± 110	306.5 ± 143	144 – 400	*0.291*
Aspartic acid (Asp)	10.9 ± 8	17.8 ± 7	0 – 25	*0.002*
Glutamine (Gln)	617.7 ± 145	461.8 ± 202	333 – 809	*0.002*
Glutamate (Glu)	102.3 ± 51	107.2 ± 64	24 – 110	*0.625* ^*∗*^

^*∗*^Mann–Whitney-*U* test was used.

## Data Availability

The SPSS statistics data document used to support the findings of this study are available from the corresponding author upon reasonable request.
